# Case Report: Late-Onset Lennox-Gastaut Syndrome Treated With Stereotactic Electroencephalography-Guided Radiofrequency Thermocoagulation Before Craniotomy

**DOI:** 10.3389/fneur.2022.857767

**Published:** 2022-06-20

**Authors:** Sixian Li, Xiaodong Cai, Chen Yao, Yuanqing Wang, Xiaohua Xiao, Huafeng Yang, Yi Yao, Lei Chen

**Affiliations:** ^1^Department of Neurosurgery, Shenzhen Second People's Hospital, The First Affiliated Hospital of Shenzhen University, Shenzhen, China; ^2^Department of Functional Neurosurgery, Xiamen Humanity Hospital, Xiamen, China; ^3^Department of Neurology, West China Hospital in Sichuan University. Chengdu, China

**Keywords:** Case Report, refractory epilepsy, late-onset Lennox-Gastaut syndrome, stereotactic electroencephalography, radiofrequency thermocoagulation

## Abstract

The onset of Lennox-Gastaut syndrome (LGS), a severe epilepsy syndrome, is typically before 8 years of age. Late-onset LGS (with onset in adolescence and adulthood) is relatively rare clinically and has some differences from classical LGS. Herein, we describe the case of a patient with late-onset LGS and provide a literature review of such cases. The patient had focal epilepsy onset at 8 years of age. After a 9-year evolution, he suffered seizures of different types and had a diagnosis of late-onset LGS. Drug treatment was ineffective. Nothing was found on stereotactic electroencephalography (SEEG) and magnetic resonance imaging (MRI) during the course of the disease. After the second presurgical evaluation, we found a suspicious focus on high-resolution structural MRI which was verified by SEEG at last. After SEEG-guided radiofrequency thermocoagulation (RFTC), his seizures were controlled, and his cognitive function and quality of living clearly improved. However, his seizures recurred 2 years later, and he underwent left occipital resection. Thereafter, his seizures have been controlled until now. This case emphasizes the importance of high-resolution structural MRI in the treatment of LGS. Furthermore, it suggests that late-onset LGS may be caused by focal lesions and evolve from focal epilepsy. Thus, characterizing the clinical symptoms and performing individualized electroencephalographic follow-up are both very important. Additionally, the clinical outcome in this case implies the value and limitations of RFTC in patients with epilepsy and a clear focal lesion. Moreover, this case further supports differences between late-onset and classical LGS in terms of clinical manifestation, cognitive changes, prognosis, and treatment.

## Introduction

Lennox-Gastaut Syndrome (LGS) is a severe epilepsy syndrome, with onset typically before 8 years of age and comprising a variety of seizure types. LGS is often accompanied by specific changes on electroencephalography (EEG) and cognitive impairment. Herein, we describe a patient with late-onset LGS who had onset after 8 years of age. For a comprehensive analysis, previous cases of late-onset LGS were reviewed and compared to this case.

## Case Presentation

### Patient Information

A 19-year-old man first experienced a generalized seizure attack without any cause at 8 years of age. Subsequently, he sometimes complained of bright or colorful flashing lights, blurry vision, and distance changes in both visual fields before seizures, especially on the right side. The visual auras usually lasted several min and sometimes occurred alone. At 17 years of age, he experienced transient nodding attacks, frequently without an aura. At 18 years of age, dropping attacks occurred frequently, with accompanying gripped hands and slight forearm flexion. After the attacks, he could stand by himself without an aura or a memory. The nodding and dropping attacks could occur 2–10 times in 4–5 days and in a series. He was diagnosed with refractory epilepsy and underwent presurgical evaluation at another hospital. Magnetic resonance imaging (MRI) scans were considered negative, and he was monitored by stereotactic electroencephalography (SEEG) focusing on the temporal, parietal, and occipital (TPO) regions, especially on the left side, but without lateralization or localization. Although he underwent vagal nerve stimulation (VNS) using five types of antiepileptic drugs, there was no significant improvement. Thus, he was admitted to our hospital for further treatment.

### Clinical Findings

**Figure 1:**
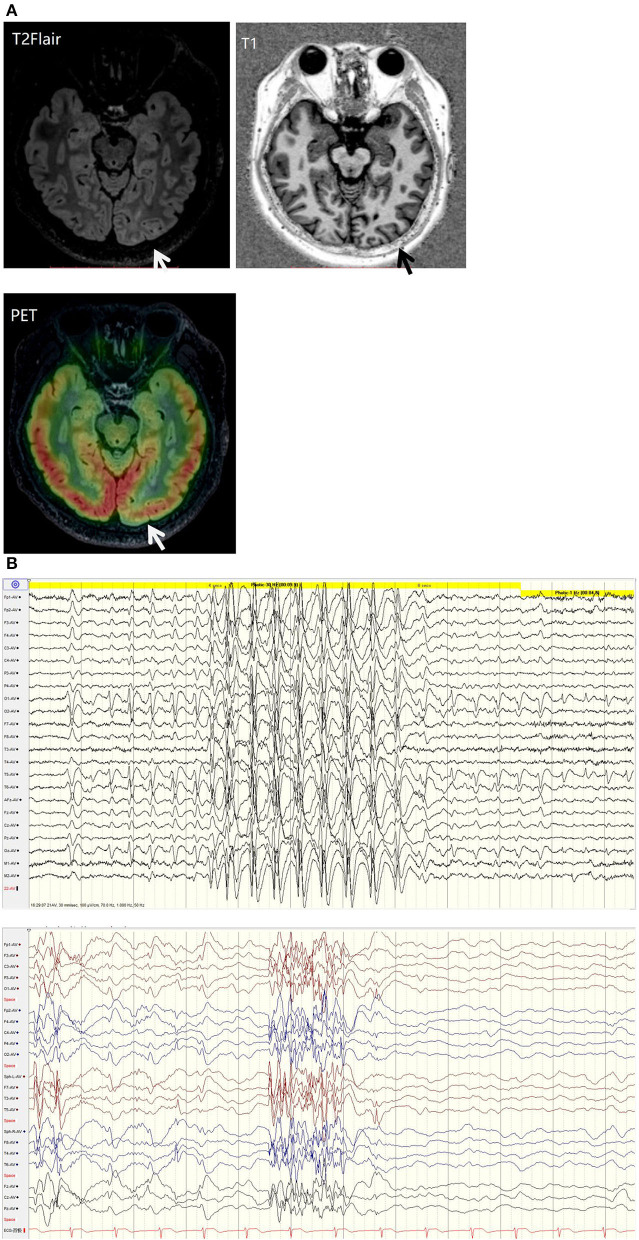
**(A)** T2 fluid-attenuated inversion recovery (T2-FLAIR) magnetic resonance imaging showing a suspiciously unclear gray-white matter boundary around the left calcarine sulcus (white arrow). T1 magnetic resonance imaging showing a suspiciously unclear gray-white matter boundary around the left calcarine sulcus (black arrow). Positive emission tomography showing low metabolism at the cortex around the left calcarine sulcus (white arrow). **(B)** EEG background: generalized 2-2.5 Hz slow spike waves (SSWs) during wakefulness and generalized paroxysmal fast activity (GPFA) during sleep.

**Figure 2:**
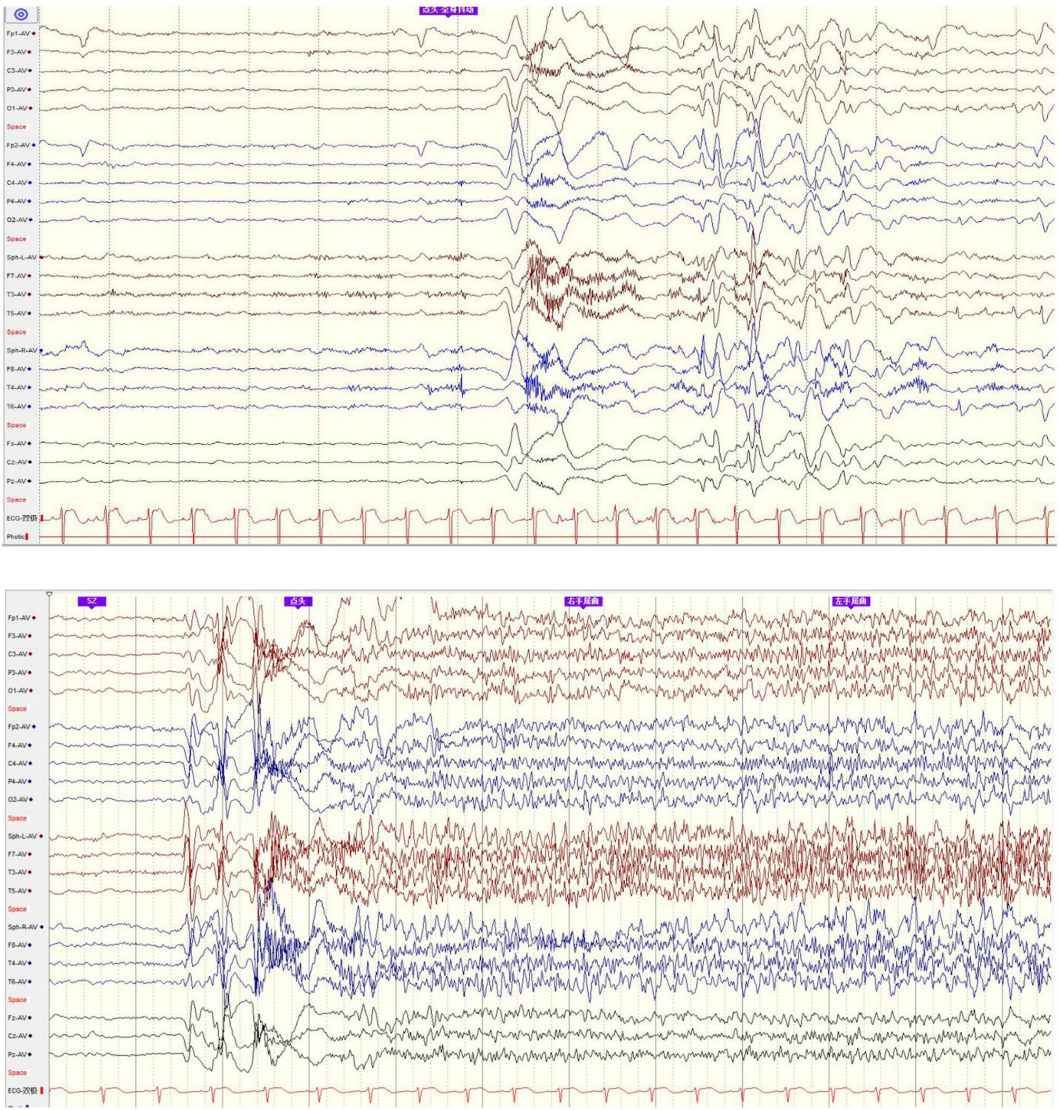
EEG onset: epileptic spasm and tonic seizure involved general EEG onsets with little localization value.

His family, perinatal, and growth history, and physical examination results were normal. Wakeful background on video electroencephalography showed 9–10 Hz alpha rhythm in bilateral occipital regions. Interictal EEG showed generalized 2–2.5 Hz slow spike waves (SSWs) during wakefulness and generalized paroxysmal fast activities (GPFAs) during sleep. There were also multifocal independent spikes, especially in the bilateral occipital and posterior temporal regions where the left side was more predominant than the right. Compared with SSWs and occipital discharges, GPFA was more prominent, followed by occipital discharges. Epileptic spasms, tonic seizures, and atypical absence were recorded, which showed EEG onsets with a little localization value. MRI showed a suspiciously unclear gray-white matter boundary around the left calcarine sulcus (Figures 1, 2), which also showed low metabolism on positron emission tomography (Figure 1). Neuropsychological tests showed moderate cognitive impairment. No abnormalities were found on genetic testing and perimetry. Accordingly, the clinic diagnoses were as follows: refractory epilepsy, late-onset LGS, possible onset zone at the left occipital cortex, and postoperative VNS.

### Therapeutic Intervention

We analyzed his previous SEEG even though the former hospital considered the SEEG result without lateralization or localization value and performed the VNS, but we found there were rhythmic discharges mainly in the left TPO region, which also had the most obviously electrical evolution during tonic and spasm seizures onset. Combining the imaging finding, we considered the seizure onset was most likely to be on the left TPO region and re-inserted six electrodes in total, of which five were focused on left TPO region and one was focused on the left hippocampus. During the SEEG monitoring, there were plentifully rhythmic interictal discharges in the left occipital region and higher frequency of fast activities during spasm and tonic seizures onset, which verified the left occipital lesion as the epileptogenic zone. Radiofrequency thermocoagulation (RFTC) was performed on this lesion and the surrounding region, with five electrodes and 17 contacts in total.

### Follow-Up and Outcomes

Following the treatment with RFTC, he did not experience seizures for more than 1 year, but the right visual hemianopsia remained. There were a few spikes on EEG (only one during a 24-h test), and his cognition improved. However, he again experienced tonic seizures 2 years after the RFTC; thus, he underwent left occipital resection. The pathology indicated micro-malformation of cortical development (mMCD) (1, 2). As yet, he has remained seizure-free for 1 year following the occipitectomy.

## Discussion

The onset of LGS is typically before 8 years of age, with a peak at 4–5 years (3). Late-onset LGS, occurring in adolescents or adults (> 8 years old), is relatively rare and accounts for 10–15% of all LGS cases (4, 5). Thus, we collected data on all cases of late-onset LGS that have been reported since 1991; the 42 identified cases are summarized in Table 1 (6–13). The age of seizure onset ranged from 6 weeks to 28 years, while the age at LGS diagnosis ranged from 8 to 64 years, The lag time between seizure onset and LGS diagnosis is mostly due to ictal semiology and electroclinical findings not having been fulfilled, suggesting that electroclinical changes in LGS follow a gradual evolutionary process, as in this case. It generally takes 1–2 years from seizure onset to a definite diagnosis of LGS (14, 15) because of differences in etiology, clinical characteristics, and disease progression between patients. This emphasizes the importance of individual electroclinical follow-up for early diagnosis of LGS. Additionally, for patients with a definite LGS diagnosis, a retrospective analysis of the characteristics of their electroclinical evolution is necessary and useful for precise treatment.

**Table 1:** Clinical characteristics, EEG and imaging findings, and other case details in 42 cases of late-onset Lennox-Gastaut Syndrome (LGS).

**NO**.	**Sex**	**Seizure onset** **(age, y)**	**Diagnosis (age, y)**	**Seizure types**	**Cognition**	**Living independently**	**Neurologic exam**	**EEG background**	**EEG epileptiform discharges**	**Imaging**	**Etiology**	**AEDS** **(≥3)**	**VNS**	**Prognosis**
1	M	14	22	T, AA, GTC	N	N	N	N	GPFA, SSWs	N	/	Y	N	/
2	M	12	17	T, AA, P	N	N	N	N	GPFA, SSWs, MS	N	/	Y	N	/
3	M	17	46	T, P	N	N	N	N	GPFA, SSWs, MS	N	/	Y	N	/
4	M	6w	8	A, AA, T	AB	AB	VI	/	GPFA, SSWs, MS	/	Infection	Y	N	/
5	M	18m	8	A, T	AB	AB	/	/	GPFA, SSWs, MS	/	/	Y	N	Mostly controlled
6	F	9	19	A, GTC	mild	AB	/	Slow	SSWs	/	/	Y	Y	Mostly controlled
7	F	28	28	AA, GTC	mild	AB	LH	Slow	SSWs	Right hemispheric stroke	Stroke	Y	Y	/
8	F	7	32	A, P, SI	AB	AB	/	Slow	SSWs	/	/	Y	Y	/
9	F	13	27	A, T, AA, GTC	N	N	N	N	GPFA, SSWs, GS	N	/	Y	N	/
10	F	12	49	T, AA, A, GTC	N	N	N	N	GPFA, SSWs, GS	N	/	Y	N	/
11	M	17	61	T, AA, M, GTC	N	N	N	N	GPFA, SSWs, GS	N	/	Y	N	/
12	F	12	44	T, AA, A, GTC	N	N	N	N	GPFA, SSWs, GS	N	/	Y	N	/
13	F	16	41	T, A, M, GTC	N	N	N	N	GPFA, SSWs, GS	N	/	Y	N	/
14	M	11	40	T, AA, A, GTC	N	N	N	N	GPFA, SSWs, GS	N	/	Y	N	/
15	F	17	22	T, AA, A, GTC	N	N	N	Slow	GPFA, SSWs, GS	N	/	Y	N	/
16	M	13	26	T, AA, A, GTC	N	N	N	Slow	GPFA, SSWs, GS	N	/	Y	N	/
17	/	20	20	T, AA, A, M, GTC	AB	/	/	/	GPFA, SSWs, MS	N	Brain injury	Y	N	/
18	F	16	16	T, A, GTC	N	/	N	/	GPFA, SSWs, GS	Low-lying cerebellar tonsils just below the foramen magnum level	/	Y	Y	Encephalopathy, DS
19	M	5	32	T, AA, A, GTC	N	/	N	/	GPFA, SSWs, GS	Small probable arachnoid cyst in the left frontal region	/	Y	Y	DS
20	F	9	14	T, AA, A, M, GTC	AB	/	N	/	GPFA, SSWs, GS	Modest ventricular dilatation	ALL with MTX	Y	N	Encephalopathy, DS
21	F	15	26	T, AA, A, M, GTC	N	/	N	/	GPFA, SSWs, GS	N	/	Y	Y	Encephalopathy, DS, SUDEP
22	M	19	19	T, A, GTC	AB	/	N	/	GPFA, SSWs, GS	N	Infection	Y	N	DS
23	F	15	15	T, AA, A, GTC	AB	/	N	/	GPFA, SSWs, GS	Single non-enhancing 6 mm focus in the paramedian inferior left cerebellum	/	Y	Y	VNS partially effective, DS
24	F	11	11	T, AA, A, GTC	N	/	N	/	GPFA, SSWs, GS	Gray matter of heterotopia with transmantle dysplasia	ALL with MTX; MCD	Y	Y	Encephalopathy, DS
25	F	11	11	T, AA, A, GTC	N	/	N	/	GPFA, SSWs, GS	Cerebellar atrophy	/	Y	Y	Encephalopathy, DS
26	F	11	11	T, A, GTC	AB	/	N	/	GPFA, SSWs, GS	Pachygyria, diffuse bilateral lissencephaly and band heterotopia	MCD	Y	Y	DS
27	F	10	10	T, AA, A, M, GTC	AB	/	N	/	GPFA, SSWs, GS	Mild cerebellar and cortical cerebral atrophy	/	Y	N	DS
28	F	14	64	AA, GTC, A	N	/	N	N	GPFA, SSWs, MS	N	/	Y	N	/
29	F	9	27	T, AA	AB	/	N	/	GPFA, SSWs	Moderate diffuse atrophy	trisomy 21	Y	N	Weekly seizures, SUDEP
30	F	8	12	T, AA, A	AB	/	N	/	GPFA, SSWs	/	trisomy 21	Y	N	DS
31	M	11	14	T, AA, A	AB	/	N	/	GPFA, SSWs	Multiple small subcortical calcification	trisomy 21	Y	N	DS
32	F	12	30	T, AA	AB	/	N	/	GPFA, SSWs	N	trisomy 21	Y	N	Seizures decreased
33	F	8	33	AA, A	AB	/	N	/	GPFA, SSWs	/	trisomy 21	Y	N	Weekly seizures, SUDEP
34	M	5	30	T, GTC	AB	/	LH	/	GPFA, SSWs	/	trisomy 21	Y	N	DS
35	M	12	17	T, AA	AB	/	N	/	GPFA, SSWs	Mild diffuse atrophy	trisomy 21	Y	N	DS
36	M	6	36	T, AA, P, GTC	AB	/	N	/	GPFA, SSWs	/	trisomy 21	Y	N	DS
37	M	16	23	T, AA, GTC	AB	/	N	/	GPFA, SSWs	Moderate diffuse atrophy	trisomy 21	Y	N	DS
38	M	11	12	T, M, P	AB	/	N	/	GPFA, SSWs	Mild diffuse atrophy	trisomy 21	Y	N	DS
39	M	7	11	T, AA, A, GTC	AB	/	N	/	GPFA, SSWs	N	trisomy 21	Y	N	Weekly or monthly seizures
40	M	10	43	T	AB	/	N	/	GPFA, SSWs, MS	Dilation cortical sulci, calcification globus pallidus	trisomy 21	Y	N	DS
41	M	7	34	T, AA	AB	/	N	/	GPFA, SSWs	Mild diffuse atrophy	trisomy 21	Y	N	DS
42	M	9	30	T, AA, GTC	AB	/	slight cerebellar sign	Slow	GPFA, SSWs, MS	Asymmetrical cystic lesions in bilateral parietooccipital regions	Perinatal injury	/	/	/

*/, information not available; M, male; F, female; y, year; w, week; m, month; N, normal; AB, abnormal; A, atonic; AA, atypical absence; T, tonic; IS, infantile spasm; GTC, generalized tonic-clonic; P, partial; SI, startle induced; M, myoclonic; VI, visual impairment; LH, left hemiparesis; GPFA, generalized paroxysmal fast activity; SSWs, slow spike waves; MS, multifocal spikes; GS, generalized spike; ALL, acute lymphoblastic leukemia; MTX, methotrexate; MCD, malformations of cortical development; AEDs, antiepileptic drugs; VNS, vagal nerve stimulation; Y, yes, N, none; SUDEP, sudden unexpected death in epilepsy; DS, daily seizures*.

Among the identified 42 cases of late-onset LGS, 13 showed chromosome variations. Of the 29 remaining cases, 17 showed normal cognition and had a disease course of 5–32 years, while 12 showed cognitive impairment (mild cognitive impairment in two cases) and a course of 5–25 years. This indicates that unlike early-onset LGS, cognitive function, and daily viability are relatively preserved in late-onset LGS; additionally, cognitive function is not significantly related to disease course (4, 5, 8). This case had a disease course of 9 years, but the patient's cognitive function had only moderately declined. Furthermore, neurological examination results were mentioned in 38 of the 42 cases, among which four patients had abnormal neurological signs related to intracranial infection, cerebral infarction, perinatal injury, etc. Additionally, the EEG background was mentioned in 16 of the 42 cases, among which 10 showed normal EEG background, and six showed nonspecific slow waves. These findings suggest that late-onset LGS mostly shows no positive neurological signs or special abnormalities in the EEG background, as observed in this case.

Among the 42 cases, MRI findings were negative in 17, nonspecific or subcortical changes were found in 12, local cortical lesions were found in 5, and MRI findings were not mentioned in the remaining 8, suggesting that the cortical structure in late-onset LGS mostly showed no specific changes. However, this substantially differs from previous reports on classical LGS, which indicate that more than 2/3 of patients show cortical structural lesions on MRI (3, 16), including focal, multifocal, and diffuse abnormalities, with static pathological lesions predominating (e.g., focal cortical dysplasia), while progressive or metabolic lesions are relatively rare (17, 18). This may reflect a difference between early- and late-onset LGS, but it may also be related to a case inclusion bias and skill in image reading; thus, this potential distinction needs to be confirmed by accumulating more cases. However, the progression of neuroimaging technology and the quality of MRI scans may also contribute to focal structural lesion identification. In this case, nothing was found in the initial clinical work-up, and the epileptic focus was found during the re-evaluation process by high-resolution structural MRI and later confirmed by SEEG and RFTC, indicating the important role of high-resolution structural MRI in the etiological diagnosis and treatment of late-onset LGS. Routine MRI should include three-dimensional T1, T2 FLAIR, and coronal thin-layer scanning of the long axis of the vertical hippocampus (3).

In the 29 cases without chromosome variations, the prognosis was not mentioned in 6 cases, 11 cases had independent viability, 2 cases had well-controlled seizures, and the remaining 10 cases had a poor prognosis, including frequent seizures, encephalopathy, sudden unexpected death in epilepsy, etc. This indicates that the prognosis of late-onset patients is relatively good. Furthermore, although most of the 42 identified cases had tried more than three antiepileptic drugs, none underwent surgery, which may be related to an unclear etiology. In total, 10 cases had tried VNS, which was clearly mentioned as effective in only 1 case, suggesting that VNS has a poor effect on late-onset LGS, as observed in this case, which showed obvious improvement after surgery, suggesting the effectiveness and necessity of early surgery for patients with late-onset LGS with definite focal lesions (15, 18).

For 1 year after the RFTC, not only were the patient's seizures well controlled, but his cognition improved and the EEG spikes “calmed down” gradually for 2 years. Such changes were first mentioned in 1979 (19). These observations not only suggest that electroclinical changes in late-onset LGS are the result of secondary alterations in brain networks (20), but also confirm the efficacy of RFTC. However, his seizure recurred and he eventually underwent left occipital resection and has been seizure free until now. This indicated the limited efficacy and scope of RFTC compared to craniotomy in lesion damage and pathological network disconnection in some cases with accurate lesion targets, which may be the main reason explaining the low rate of seizure-free cases with RFTC (21). However, the improvement of clinical outcomes after RFTC may further suggest the correct location of epileptic foci and good prognosis after craniotomy, as in this case. Nevertheless, RFTC is superior in some small foci that can be located precisely and destroyed completely but are inaccessible by surgery, and has the advantages of less damage to brain function, fewer complications, and faster postoperative recovery (22, 23).

In summary, LGS, with onset after 8 years of age known as late-onset LGS, may be caused by focal lesions. Individualized follow-up is necessary. Late-onset LGS and classical LGS have some differences in terms of clinical manifestation, cognitive changes, prognosis, and treatment. MRI is important in determining the etiology of late-onset LGS and is useful for early treatment. Comparing to craniotomy, RFTC is limited and less effective in lesion damage. However, clinical improvement after RFTC would support the appropriate identification of seizure onset zone location indicated by SEEG results and therefore help with surgical plan for cortical resection.

## Patient Perspective

We spent a lot of money and time in the treatment of our son's disease before the second evaluation. After the RFTC, our son's seizures were miraculously controlled and he could go to school again. This was beyond our expectations.

## Data Availability Statement

The original contributions presented in the study are included in the article/supplementary material, further inquiries can be directed to the corresponding author/s.

## Ethics Statement

Written informed consent was obtained from the individual(s) for the publication of any potentially identifiable images or data included in this article.

## Author Contributions

All authors listed have made a substantial, direct, and intellectual contribution to the work and approved it for publication.

## Conflict of Interest

The authors declare that the research was conducted in the absence of any commercial or financial relationships that could be construed as a potential conflict of interest.

## Publisher's Note

All claims expressed in this article are solely those of the authors and do not necessarily represent those of their affiliated organizations, or those of the publisher, the editors and the reviewers. Any product that may be evaluated in this article, or claim that may be made by its manufacturer, is not guaranteed or endorsed by the publisher.
